# Early structural valve deterioration of a pulmonary position Inspiris Resilia valve requiring redo pulmonary and tricuspid valve replacement

**DOI:** 10.1093/jscr/rjaf898

**Published:** 2025-11-12

**Authors:** Akito Inoue, Ryohei Ushioda, Hidenobu Akamatsu, Tasuku Kawarabayashi, Jeonga Lee, Jun Maruoka, Kentaro Shirakura, Yuki Setogawa, Ryo Okubo, Hiroyuki Miyamoto, Aina Hirofuji, Shogo Takahashi, Daisuke Takeyoshi, Shingo Kunioka, Hiroyuki Kamiya

**Affiliations:** Department of Cardiac Surgery, Asahikawa Medical University, midorigaoka 1-1-1, Asahikawa, Hokkaido, 078-8510, Japan; Department of Cardiac Surgery, Asahikawa Medical University, midorigaoka 1-1-1, Asahikawa, Hokkaido, 078-8510, Japan; Department of Cardiac Surgery, Asahikawa Medical University, midorigaoka 1-1-1, Asahikawa, Hokkaido, 078-8510, Japan; Department of Cardiac Surgery, Asahikawa Medical University, midorigaoka 1-1-1, Asahikawa, Hokkaido, 078-8510, Japan; Department of Cardiac Surgery, Asahikawa Medical University, midorigaoka 1-1-1, Asahikawa, Hokkaido, 078-8510, Japan; Department of Cardiac Surgery, Asahikawa Medical University, midorigaoka 1-1-1, Asahikawa, Hokkaido, 078-8510, Japan; Department of Cardiac Surgery, Asahikawa Medical University, midorigaoka 1-1-1, Asahikawa, Hokkaido, 078-8510, Japan; Department of Cardiac Surgery, Asahikawa Medical University, midorigaoka 1-1-1, Asahikawa, Hokkaido, 078-8510, Japan; Department of Cardiac Surgery, Asahikawa Medical University, midorigaoka 1-1-1, Asahikawa, Hokkaido, 078-8510, Japan; Department of Cardiac Surgery, Asahikawa Medical University, midorigaoka 1-1-1, Asahikawa, Hokkaido, 078-8510, Japan; Department of Cardiac Surgery, Asahikawa Medical University, midorigaoka 1-1-1, Asahikawa, Hokkaido, 078-8510, Japan; Department of Cardiac Surgery, Asahikawa Medical University, midorigaoka 1-1-1, Asahikawa, Hokkaido, 078-8510, Japan; Department of Cardiac Surgery, Asahikawa Medical University, midorigaoka 1-1-1, Asahikawa, Hokkaido, 078-8510, Japan; Department of Cardiac Surgery, Asahikawa Medical University, midorigaoka 1-1-1, Asahikawa, Hokkaido, 078-8510, Japan; Department of Cardiac Surgery, Asahikawa Medical University, midorigaoka 1-1-1, Asahikawa, Hokkaido, 078-8510, Japan

**Keywords:** pulmonary valve replacement, Inspiris Resilia, structural valve deterioration, tricuspid valve replacement, redo cardiac surgery

## Abstract

We report a 50-year-old woman with pulmonary and tricuspid valve regurgitation who required redo pulmonary and tricuspid valve replacement (PVR, TVR). At age 8, she underwent right ventricular outflow tract reconstruction for pulmonary stenosis, and at 44 years she had PVR with a 19 mm Inspiris Resilia bioprosthesis and tricuspid annuloplasty with a 28 mm Physio ring. Three years later, she presented with palpitations and syncope. Echocardiography revealed severe tricuspid regurgitation and moderate pulmonary regurgitation due to dysfunction of a prosthetic leaflet. Redo PVR with a 29 mm Inspiris Resilia valve and TVR with a 27 mm Mitris valve was performed. Early structural valve deterioration of the initial Inspiris prosthesis was suspected. Our experience suggests that using a larger prosthesis may mitigate early degeneration and preserve the option of future transcatheter PVR.

## Introduction

Pulmonary valve replacement (PVR) is a common late intervention for patients with congenital heart disease who have undergone prior right ventricular outflow tract (RVOT) reconstruction. Although the Inspiris Resilia bioprosthesis demonstrates excellent durability in the aortic position, recent reports have described early structural valve deterioration (SVD) when implanted in the pulmonary position, raising concerns about its performance in the low-pressure right-sided circulation. However, the optimal choice of prosthetic valve for PVR remains uncertain, as no biological substitute—including bovine pericardial valves, porcine valves, or homografts—has shown clear superiority in long-term durability.

In this report, we describe a patient who developed early SVD of an Inspiris Resilia pulmonary valve. Based on this experience, we emphasize the importance of implanting the largest feasible prosthesis at the time of surgery to minimize transvalvular gradients, potentially slow degeneration, and preserve the option of future transcatheter PVR.

## Case report

**Figure 1:**
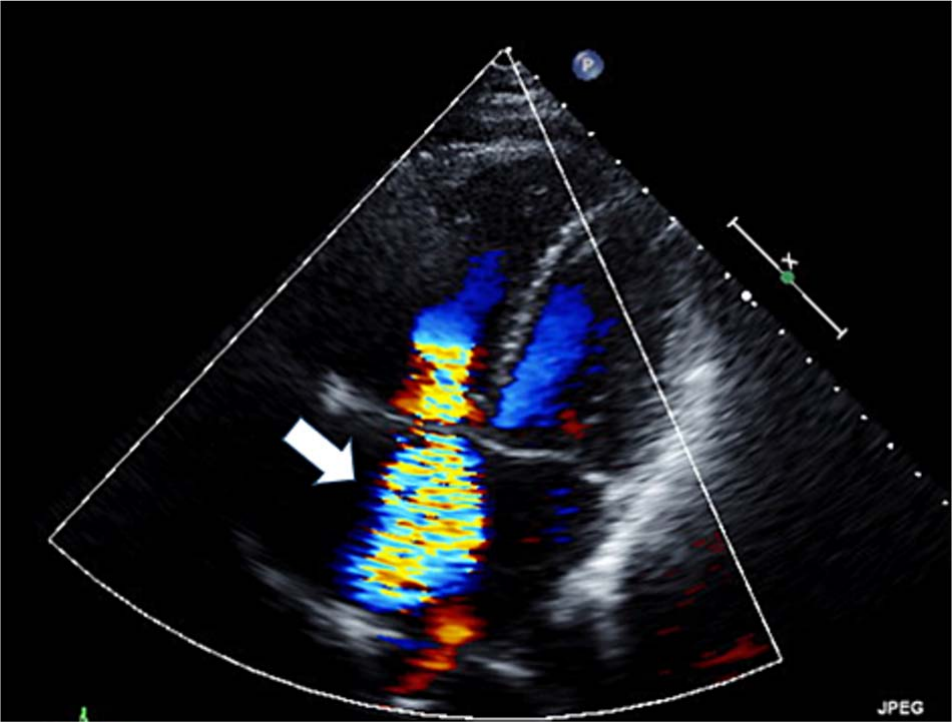
Preoperative transthoracic echocardiography: severe tricuspid regurgitation. The regurgitant jet is indicated by the white arrow.

**Figure 2:**
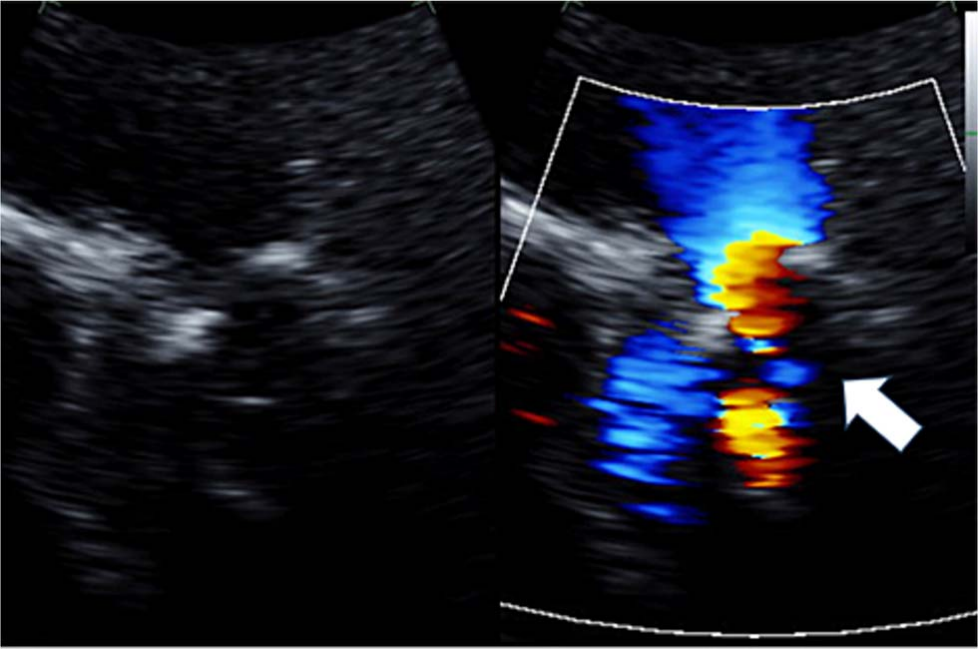
Preoperative transthoracic echocardiography: moderate pulmonary regurgitation. The regurgitant jet is indicated by the white arrow.

The patient was a 50-year-old female with pulmonary (PV) and tricuspid valve (TV) regurgitation. At 8 years old, she received RVOT reconstruction due to PV stenosis. When she was 44 years old, transthoracic echocardiography revealed PV regurgitation and PVR (INSPIRIS 19 mm; Edwards Lifesciences Corporation, Irvine, CA, USA) and tricuspid annuloplasty (Physio annuloplasty ring 28 mm; Edwards Lifesciences, Irvine, CA, USA) was performed. After 3 years of this operation, she gradually became complained of palpitation and syncope. Transthoracic echocardiography revealed severe tricuspid regurgitation due to leaflet tethering and moderate pulmonary regurgitation caused by dysfunction of one of the tissue valve leaflets (Figs 1 and 2). Cardiac magnetic resonance imaging showed right ventricular enlargement (right ventricular end-diastolic volume index; 97.5 ml/m^2^). Finally, we decided to perform a redo PV and TV replacement.

**Figure 3:**
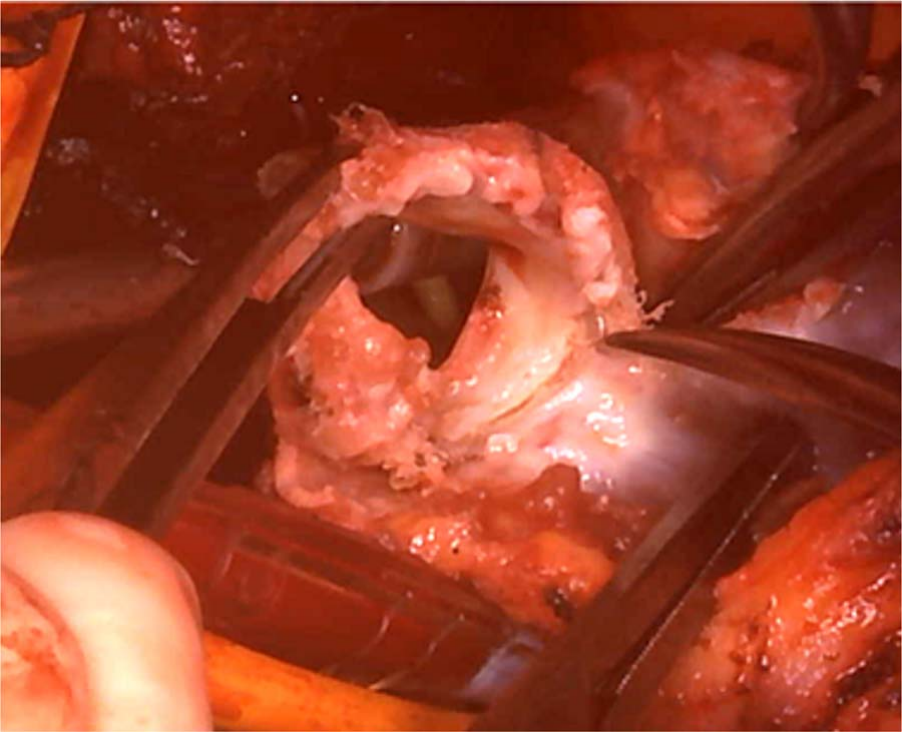
Intraoperative view: the pulmonary artery and right ventricular outflow tract opened with the previously implanted pulmonary valve explanted.

Cardiopulmonary bypass was established with ascending aorta and bicaval cannulation. The right atrium was incised, the previous TV ring was removed, and a tissue valve (MITRIS 27 mm; Edwards Lifesciences Corporation, Irvine, CA, USA) was implanted. The pulmonary artery was opened to the RVOT, and the previously implanted valve was removed (Fig. 3). The valve leaflet was not calcified but was thickened and shortened, showing a restricted opening. PVR was performed again with a 29 mm INSPIRIS valve. RVOT was closed using Gore-Tex artificial vessels graft (expanded polytetrafluoroethylene; W. L. Gore & Associates, Flagstaff, AZ, USA) for patching.

She was extubated on the first postoperative day but was reintubated and performed a tracheostomy due to respiratory failure. After the tracheotomy tube was removed, she was transferred to another hospital at postoperative 80 days. After 3 months of postoperative echo showed no paravalvular and transvalvular leakage. At 3 years postoperatively, the patient remains in good health and continues to lead an uneventful life.

## Discussion

Early SVD of the Inspiris Resilia valve in the pulmonary position has been increasingly documented in recent years. Said *et al*. retrospectively analyzed 27 patients who underwent PVR using the Inspiris Resilia valve and found that nearly half of the patients implanted with standard surgical technique developed new-onset prosthetic regurgitation during early follow-up, suggesting that the valve may be unsuitable for low-pressure right-sided circulation or that modifications to the implantation technique may be necessary [1]. Following this, Nguyen *et al*. demonstrated that the 2-year valve failure-free survival in patients who underwent Inspiris implantation in the native RVOT was significantly lower compared to other prostheses, with a notably higher incidence of pulmonary regurgitation (53.5% vs. 78.5%, *P* = .03) [2]. Consistent with these findings, our patient developed early valve dysfunction following Inspiris valve implantation.

However, the optimal choice of prosthetic valve for PVR remains a matter of debate. The long-term durability differences between bovine pericardial valves and porcine aortic valves are yet to be clearly established. Chen *et al*. reported no significant difference in reintervention rates between bovine pericardial and porcine bioprosthetic valves during midterm follow-up [3]. While homografts and Contegra conduits (bovine jugular vein grafts) are commonly used in Western countries, they are difficult to obtain in Japan due to regulatory and supply limitations. Given these circumstances, we consider there to be no decisive advantage of one biological material over another based on current evidence.

In this case, we again selected the Inspiris Resilia valve, as used in the initial surgery, but opted for a larger 29 mm size. This decision was informed by findings from the aortic valve literature, which demonstrate that small surgical bioprostheses (≤21 mm) are independently associated with an increased risk of SVD as reported by Flameng *et al*. [4]. We believe that this principle also applies to the pulmonary position, where the use of a larger valve may help reduce the risk of early SVD.

Furthermore, another important reason for selecting a large-diameter valve was to ensure eligibility for future transcatheter PVR. Recent advances in TPVR have expanded treatment options for patients with RVOT dysfunction. However, as Giordano and colleagues highlight, current transcatheter PVR devices, including the Melody and Sapien valves, remain constrained by anatomical factors such as landing zone diameter and morphology [5]. Consequently, ensuring an adequate surgical annulus at the time of initial PVR is critical to facilitate future percutaneous interventions and to optimize long-term outcomes.

In summary, although an ideal prosthetic valve for PVR has yet to be established, our experience suggests that the use of a large-diameter prosthesis is a practical strategy to reduce the risk of early degeneration and to preserve future therapeutic options, including transcatheter techniques.
